# Arterial-embolic Strokes and Painless Vision Loss Due to Phase II Aortitis and Giant Cell Arteritis: A Case Report

**DOI:** 10.5811/cpcem.2021.2.51143

**Published:** 2021-04-19

**Authors:** Kaitlin Endres, Omar Anjum, Nicholas Costain

**Affiliations:** * University of Ottawa, Department of Medicine, Ottawa, Ontario, Canada; † The Ottawa Hospital, Department of Emergency Medicine, Ottawa, Ontario, Canada

**Keywords:** aortitis, giant cell arteritis, arterial-embolic stroke, painless vision loss

## Abstract

**Introduction:**

Aortitis refers to abnormal inflammation of the aorta, most commonly caused by giant cell arteritis (GCA). Herein, we present a 57-year-old female with aortitis and arterial-embolic strokes secondary to GCA.

**Case Report:**

Our patient presented to the emergency department following an episode of transient, monocular, painless vision loss. Computed tomography angiogram head and neck demonstrated phase II aortitis, and magnetic resonance imaging revealed evidence of arterial-embolic strokes.

**Conclusion:**

Cerebrovascular accident is a rare complication of large-vessel vasculitis and can occur due to multiple underlying etiologies including intracranial vasculitis, aortic branch proximal occlusion, or arterial-embolic stroke.

## INTRODUCTION

Aortitis refers to abnormal inflammation of the aorta with the most common causes being non-infectious, such as giant cell arteritis (GCA) and Takayasu arteritis.^1^ While aortitis is rare, it can also be potentially fatal.^2^ Here we report a case of aortitis secondary to GCA, causing multiple arterial-embolic strokes in a patient presenting with painless, transient, monocular vision loss. This case is novel as our patient presented with signs of GCA as well as neurological symptoms which did not coincide with a single vascular territory. To our knowledge, this is the first case report of a patient presenting to the emergency department (ED) with evidence of arterial-embolic strokes secondary to aortitis induced by GCA.

## CASE REPORT

A 57-year-old female with a past medical history of hypothyroidism presented to the ED following an episode of sudden left-sided, painless, vision loss that resolved within 30 minutes. This episode occurred in the context of a five-month history of fatigue, 15-kilogram weight loss, periodic night sweats, and generalized subjective weakness, along with a two-month history of intermittent headaches and jaw claudication. For the prior month, she also endorsed transient neurologic deficits, predominantly weakness of the left arm and right leg. At the time of assessment, she complained of persistent left-sided hand clumsiness.

On examination, the patient’s vitals were stable. She had a normal cardiopulmonary exam. Her neurologic exam included normal cranial nerves, a normal gait and cerebellar exam, as well as normal gross sensation. Her motor exam was unremarkable except for weak elbow extension (3/5) and shoulder abduction (4/5) of the left arm. She had normal visual acuity, normal pupil size and reactivity, and intact extraocular movements. Her slit lamp exam revealed normal lids, lashes, lacrimal system, conjunctivae, cornea, and anterior chamber. Her fundoscopy was normal. Laboratory data revealed the following: leukocytosis; anemia; thrombocytosis; and elevated inflammatory markers (erythrocyte sedimentation rate [ESR]: 122 millimeters per hour (mm/hr) [normal in females 0–20 mm/hr], and C-reactive protein [CRP]: 119.4 milligrams per liter (mg/L) [normal: ≤10 mg/L), overall suspicious for an underlying reactive process. Due to concern for concomitant cerebrovascular accident (CVA), a computed tomography (CT) head and CT angiogram (CTA) head and neck were performed. While initial imaging did not reveal an acute stroke, CTA showed prominent circumferential wall thickening of the descending aorta, aortic arch and great vessels of the neck and chest (including the left common carotid artery and left subclavian artery). Her CTA revealed no evidence of luminal narrowing or aneurysm formation.

The patient’s clinical picture was most consistent with large vessel vasculitis, likely GCA with ophthalmologic and possible central nervous system (CNS) involvement. In consultation with rheumatology, she was started on high-dose pulse glucocorticosteroids with methylprednisolone 500 mg intravenous daily for three days followed by prednisone 60 mg daily with a slow taper. A complete CT chest/abdomen/pelvis was performed that revealed diffuse aortic wall thickening (Image). Neurology was consulted, and a magnetic resonance imaging (MRI) was recommended which revealed subacute cortical infarcts in the right frontal lobe, left parietal lobe, and remote lacunar infarcts. Based on expert opinions from stroke neurology and neuroradiology, these strokes were felt to be arterial-embolic in nature, secondary to aortitis.

**CPC-EM Capsule**

What do we already know about this clinical entity?

*Aortitis, or aorta inflammation, is most commonly caused by giant cell arteritis (GCA). Stroke is a rare but devastating complication of GCA*.

What makes this presentation of disease reportable?

*This is a unique case of a patient presenting to the emergency department (ED) with evidence of arterial-embolic strokes secondary to aortitis induced by GCA*.

What is the major learning point?

*Evidence of aortitis on computed tomography (CT) angiogram head/neck for routine stroke workup should prompt a CT aortogram to rule out phase III aortitis findings*.

How might this improve emergency medicine practice?

*Recognition of aortitis by emergency physicians helps ensure early consultation to specialists for management, helping prevent progression and sequelae*.

The treatment for arterial embolic stroke secondary to aortitis is management of the underlying vasculitis (ie, steroids). Anti-platelet therapy is usually included as well. The role of tissue plasminogen activator and endovascular treatment is based on consultation with stroke neurology and determination of size, location, and timing of the strokes. Ophthalmology performed a temporal artery biopsy, which ultimately revealed a diagnosis of GCA in this patient. She was discharged after five days in stable condition with no new symptoms, and with outpatient rheumatology follow-up.

## DISCUSSION

Aortitis is commonly classified into three phases. Phase I presents with non-specific signs of systemic inflammation including fever, arthralgias, and elevated acute phase reactants.^3^ Phase II involves vascular inflammation causing arterial pain and/or tenderness, and phase III involves permanent arterial wall injury from ischemia secondary to occlusion of proximal and distal aortal branches.^3^ Our patient had no intraluminal narrowing on CTA to suggest phase III aortitis; however, imaging did show diffuse and circumferential aortic wall inflammation consistent with phase II aortitis. While any evidence of aortitis should alert emergency physicians to consult specialists for management options, it is optimal to diagnose aortitis in the earliest phase possible to prevent future sequelae from subsequent phases. An additional take-home message for emergency physicians is that if there is evidence of aortitis on CTA head and neck for routine stroke workup, it is worth performing a CT aortogram to ensure no abnormal phase III findings such as intraluminal narrowing, dilation and acute aortic syndromes.

Vision loss in the setting of GCA is usually secondary to arteritic anterior ischemic optic neuropathy and, less commonly, central retinal artery occlusion. Regardless of the pathophysiology, abrupt onset of visual disturbance in suspected GCA, especially transient monocular vision loss, indicates optic nerve ischemia. Immediate management with pulse glucocorticosteroids and urgent ophthalmology consult to arrange for temporal artery biopsy are required.

The condition of GCA is classified as a vasculitis of medium- and large-sized vessels with evidence of aortitis in approximately 15% of cases.^4^ Although one of the most common causes of aortitis, GCA is rare, affecting only 18.9 out of 100,000 Americans over the age of 50 each year.^5^ Interestingly, our patient presented with virtually all classic GCA findings. Most diagnostic algorithms suggest temporal artery biopsy with high suspicion for GCA and one of the following symptoms: new headache (72% prevalence); transient monocular visual loss (32%); jaw claudication (40%); constitutional signs and symptoms (25%); or elevated ESR and/or CRP (90%)^6^.

A CVA is a rare but devastating complication of GCA, occurring in only 3–4% of GCA patients.^7^ Weakness in the setting of GCA may be due to many causes including CVA or extracranial vessel involvement.^8^ Here we highlight the potential causes for our patient’s weakness: 1) CVA in the setting of GCA is most commonly caused by stenosis of the carotid and/or vertebrobasilar arteries or alternatively; 2) intracranial vasculitis as a CNS manifestation of the systemic disease (however, neither of these explanations were supported by her imaging);^9^ 3) In the setting of phase III aortitis, GCA can cause upper and lower extremity claudication secondary to arterial occlusive disease involving aortic branch vessels and larger peripheral arteries (however, there was no evidence of pain with the patient’s extremity weakness); and 4) a rare cause of CVA in the setting of GCA is inflammation-induced aortic thrombus formation, resulting in release of emboli that cause distal occlusion. This was thought to be the most likely pathophysiology for our patient’s weakness based on expert opinions from stroke neurology and neuroradiology given the MRI findings of multiple subacute cortical and lacunar infarcts.

## CONCLUSION

This case presentation highlights several important learning points on aortitis in the setting of GCA for emergency physicians. There are three distinct phases of aortitis which can be distinguished based on differences in signs and symptoms and imaging findings. Vision loss in the setting of suspected GCA necessitates pulse glucocorticosteroids before arranging for temporal artery biopsy in order to prevent progression to bilateral or permanent vision loss. A CVA in the setting of large-vessel vasculitis can be due to multiple causes including intracranial vasculopathy or vasculitis, proximal occlusion of aortic branches (phase III aortitis) or, more rarely, arterial-embolic stroke.

## Figures and Tables

**Image:**
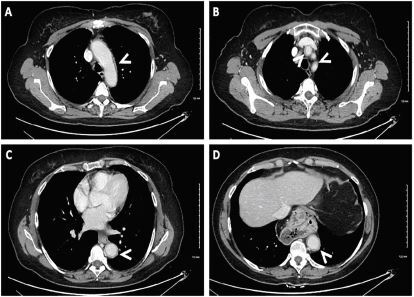
Axial views of computed tomography chest and abdomen demonstrating prominent wall thickening of the aorta (white arrows). (A) aortic arch, (B) left common carotid artery and left subclavian artery, and (C and D) descending aorta which all demonstrate diffuse and circumferential wall thickening without luminal narrowing, suggestive of phase II aortitis.

## References

[1] Gornik HL, Creager MA (2008). Aortitis. Circulation.

[2] Ostberg G (1971). Temporal arteritis in a large necropsy series. Ann Rheum Dis.

[3] Restrepo CS, Ocazionez D, Suri R (2011). Aortitis: imaging spectrum of the infectious and inflammatory conditions of the aorta. Radiographics.

[4] Bossert M, Prati C, Balblanc JC (2011). Aortic involvement in giant cell arteritis: current data. Joint Bone Spine.

[5] Babigumira JB, Li M, Boudreau DM (2017). Estimating the cost of illness of giant cell arteritis in the United States. Rheumatol Ther.

[6] Zhang Y, Wang D, Yin Y (2019). Clinical comparisons of patients with giant cell arteritis with versus without fever at onset. J Int Med Res.

[7] Cox BC, Fulgham JR, Klaas JP (2019). Recurrent stroke in giant cell arteritis despite immunotherapy. Neurologist.

[8] de Boysson H, Liozon E, Larivière D (2017). Giant cell arteritis-related stroke: a retrospective multicenter case-control study. J Rheumatol.

[9] Solans-Laqué R, Bosch-Gil JA, Molina-Catenario CA (2008). Stroke and multi-infarct dementia as presenting symptoms of giant cell arteritis: report of 7 cases and review of the literature. Medicine (Baltimore).

